# Permeation Flux Prediction of Vacuum Membrane Distillation Using Hybrid Machine Learning Techniques

**DOI:** 10.3390/membranes13120900

**Published:** 2023-12-05

**Authors:** Bashar H. Ismael, Faidhalrahman Khaleel, Salah S. Ibrahim, Samraa R. Khaleel, Mohamed Khalid AlOmar, Adil Masood, Mustafa M. Aljumaily, Qusay F. Alsalhy, Siti Fatin Mohd Razali, Raed A. Al-Juboori, Mohammed Majeed Hameed, Alanood A. Alsarayreh

**Affiliations:** 1 Construction and Projects Department, University of Fallujah, Fallujah 31002, Iraq; bashar.h.ismael@uofallujah.edu.iq; 2 Department of Civil Engineering, Al-Maarif University College (AUC), Ramadi 31001, Iraq; faidhalrahman.mohammed@uoa.edu.iq (F.K.); mk.alomar@uoa.edu.iq (M.K.A.); mustafa.k@uoa.edu.iq (M.M.A.); m.majeed@uoa.edu.iq (M.M.H.); 3 Membrane Technology Research Unit, Chemical Engineering Department, University of Technology, Alsena’a Street 52, Baghdad 10066, Iraq; salah.s.ibrahim@uotechnology.edu.iq (S.S.I.); samraarefat@yahoo.com (S.R.K.); 4 Department of Civil Engineering, Jamia Millia Islamia, New Delhi 110025, India; adil169375@st.jmi.ac.in; 5 Department of Civil Engineering, Faculty of Engineering and Built Environment, Universiti Kebangsaan Malaysia, Bangi 43600, Selangor, Malaysia; fatinrazali@ukm.edu.my; 6 Smart and Sustainable Township Research Centre (SUTRA), Universiti Kebangsaan Malaysia (UKM), Bangi 43600, Selangor, Malaysia; 7 Green Engineering and Net Zero Solution (GREENZ), Universiti Kebangsaan Malaysia (UKM), Bangi 43600, Selangor, Malaysia; 8 NYUAD Water Research Center, Abu Dhabi Campus, New York University, Abu Dhabi P.O. Box 129188, United Arab Emirates; 9 Water and Environmental Engineering Research Group, Department of Built Environment, Aalto University, P.O. Box 15200, FI-00076 Espoo, Finland; 10 Department of Chemical Engineering, Faculty of Engineering, Mutah University, P.O. Box 7, Karak 61710, Jordan; alanood.sar@mutah.edu.jo

**Keywords:** vacuum membrane distillation, desalination, flux pressure, machine learning, spotted hyena optimizer, global sensitivity analysis

## Abstract

Vacuum membrane distillation (VMD) has attracted increasing interest for various applications besides seawater desalination. Experimental testing of membrane technologies such as VMD on a pilot or large scale can be laborious and costly. Machine learning techniques can be a valuable tool for predicting membrane performance on such scales. In this work, a novel hybrid model was developed based on incorporating a spotted hyena optimizer (SHO) with support vector machine (SVR) to predict the flux pressure in VMD. The SVR–SHO hybrid model was validated with experimental data and benchmarked against other machine learning tools such as artificial neural networks (ANNs), classical SVR, and multiple linear regression (MLR). The results show that the SVR–SHO predicted flux pressure with high accuracy with a correlation coefficient (R) of 0.94. However, other models showed a lower prediction accuracy than SVR–SHO with R-values ranging from 0.801 to 0.902. Global sensitivity analysis was applied to interpret the obtained result, revealing that feed temperature was the most influential operating parameter on flux, with a relative importance score of 52.71 compared to 17.69, 17.16, and 14.44 for feed flowrate, vacuum pressure intensity, and feed concentration, respectively.

## 1. Introduction

The scarcity of drinking water has been a global problem for years. Desalination of seawater and brackish water has become one of the most promising methods to produce fresh water [1,2,3,4,5]. Desalination is a process in which saline water is separated into two parts, one that has a low concentration of dissolved salts, which is called fresh water, and another which has a much higher concentration of dissolved salts than the original feed water, which is referred to as brine or concentrate [6]. Traditionally, water desalination was achieved via thermal distillation; however, recent years have witnessed the climax of membrane technologies development and implementation. This has led to the widespread installation of pressure-driven membrane methods such as reverse osmosis (RO). While RO technology has proven its worth as a feasible desalination technique, it produces large quantities of brine that need to be properly treated. Membrane distillation (MD) is the only membrane technology that can handle streams with high salinity as the brine. 

MD is a thermally driven method that separates water from a saline aqueous solution using a microporous hydrophobic membrane [7,8]. The temperature difference across the membrane creates a water vapour pressure gradient that serves as a driving force for the pure water vapour transfer to the permeate side [9,10]. The transferred vapour then condensates onto a cold surface to produce pure water. MD has several advantages over other traditional separation technologies, including a large evaporation area integrated into a membrane module, a lower operating pressure than pressure-driven membrane processes, the capacity to utilize low-grade heat energy, and the ability to treat highly contaminated water [1,4]. There are four MD configurations, namely direct contact membrane distillation (DCMD), air gap membrane distillation (AGMD), sweep gas membrane distillation (SGMD), and vacuum membrane distillation (VMD) [10,11,12,13]. VMD stands out among the other MD systems due to its low heat loss and reduced temperature polarization effects [14,15]. The air in the permeate side is evacuated by applying a continuous vacuum below the equilibrium vapour pressure [16]. VMD can be used for a variety of purposes, and not only for seawater desalination, including ethanol recovery, the removal of trace pollutants, and the removal of volatile organic compounds (VOCs) from water [17,18]. Higher permeate water flux can be obtained with minor conductive heat loss across the membrane in the VMD setup [19]. As a result, VMD has received a lot of attention in the field of water treatment [20].

In the realm of membrane science and technology, developing a mathematical model that can predict membrane separation processes is an efficient process [21,22]. The models are useful in the simulation and optimization of membrane systems, resulting in more efficient and cost-effective separation process designs [23]. Artificial neural networks (ANNs) are a multivariate regression modelling technique that can deal with linear and non-linear behaviours [24,25]. This methodology, which is classified as a “black-box” model, does not require explicit statements of the physical meaning of the system or process under investigation. With a limited set of experimental runs, such models allow researchers to investigate the link between the input variables and the process’s targets or outputs [26]. Furthermore, with an appropriate design of trials, ANN models can be created simply [27].

ANN modelling has been used in forecasting the performance of various membrane technology processes [28]. During dead-end MD, an ANN model is used to simulate permeate flux as a function of mixed liquid suspended, temperature, dissolved oxygen, hydraulic retention time, transmembrane pressure, and operating time [27]. The structure-optimized single hidden layer neural network was able to accurately reproduce the dynamic behaviour of permeate flux, according to the authors. For membrane separation, ANN modelling was used. It was found that artificial neural networks may reliably predict real-world process behaviour with relatively low error, <5%. Permeate flux reduction in crossflow membranes for saline water removal was explored using ANN modelling. The ANN model was found to be capable of accurately predicting permeate flux from process factors such as transmembrane pressure, feed solution concentration, and membrane type [29]. For the transient crossflow membrane of polydispersed suspensions, the ANN model was used. Neural networks were utilized to model crossflow membranes dynamically.

According to recent review papers, the researchers focused mainly on the application of ANN in seawater desalination areas [30]. In other words, other machine learning techniques are still not explored in this field. Although the ANN is considered a robust prediction model, it needs lots of data samples to be effectively trained [31]. Conducting lots of experimental tests to obtain the data samples is often laborious, difficult, and expensive. The new trend of research in optimization problems is to apply heuristic algorithms to optimize the parameters of the predicting models and hence obtain highly accurate predictions. This work is dedicated to exploring the feasibility of one of these techniques, namely SVR–SHO, for predicting VMD performance and comparing the outcome with the results of common tools such as ANNs, classical SVR, and multiple linear regression. The work will also apply a novel global sensitivity analysis to interpret the predicted results and select the most influential parameters that have a large impact on the flux. To the author’s best knowledge, testing SVR–SHO for predicting VMD behaviour has not been investigated before, and this is the first attempt that has done that. 

## 2. Experimental Work

The VMD system was designed to collect experimental data for the sake of assessing the system’s performance by examining the impacts of operating variables (feed temperature, feed flow rate, feed concentration, and vacuum-side pressure) on permeate flux for the desalination procedure. A stainless-steel tube, 20 cm in length with a 10 mm outer diameter and a 9 mm interior diameter, was used to make the hollow fibre module, which was attached to a Swagelok tube fitting (10 mm) on each end. The hollow fibre model was selected because it has more potential for scalability compared to the commonly used flat sheet membrane in experimental setups. In the stainless-steel tube, commercial hollow fibre polypropylene (PP) [ACCUREL; PP S6/2 Membrane GmbH] membranes were glued with an epoxy resin (Euxi 50KII-hardener). To check for leaks in the membrane module or the system’s connections, distilled water was cycled for 10–15 min. A water bath was used to heat the salt solution to the required temperature. A peristaltic pump was used to circulate the salt solution through the membrane module’s lumen side. Water vapour was sucked from the shell side of the module into the condenser by a vacuum pump, and the water was collected in the glass trap. The temperature at the module’s input and output ports was recorded and stored in a computer. The feed’s hydrostatic pressure was regulated manually via a control valve so that it did not exceed the liquid entry pressure (LEP) of the membrane and did not wet the membrane pores. The following equation was used to calculate permeate flux:

(1) $$J=\frac{V*\rho }{A*t}$$

where *J* is permeate flux in (kg/m^2^.h), *V* is volume of fresh water (L), ρ is water density in (kg/L), *t* is the operational time in (h), and *A* is the membrane’s effective area (m^2^), which can be calculated applying Equation (2). *d_i_* is the internal diameter of fibre (m), and *L* is the effective length of fibre (m).

(2) $$A=n\pi d_{i}L$$

After around 20 min, the VMD process reached a steady state, and the process ran for another 5 h. Every 20 min, the measured amount of permeate was added to the feed solution to maintain the same feed levels and concentration in the conical flask. The feed temperature, feed flow rate, feed concentration, and absolute pressure were all varied in fifty tests.

## 3. Artificial Intelligence-Based Models

### 3.1. Support Vector Regression

**Figure 1:**
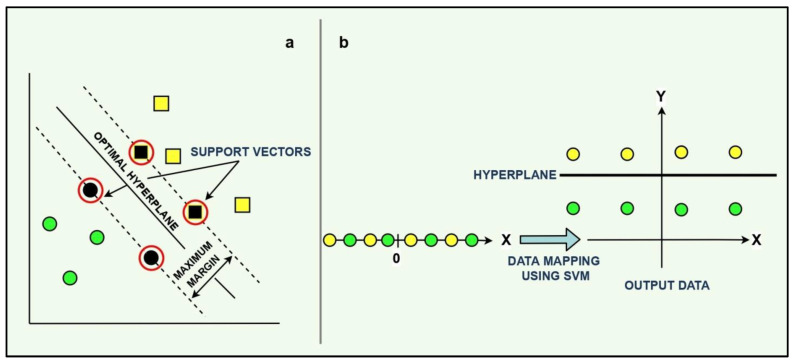
SVM features: (**a**) optimal hyperplane and the support vectors, and (**b**) mapping of data using SVM.

Support vector machines (SVMs) are sophisticated kernel-based machine learning techniques which have been successfully applied to solve a wide range of classification, numerical prediction, density estimation, and pattern recognition problems [32]. The technique uses the structural risk minimization principle to obtain an N-dimensional hyperplane with wide margins to classify the data into predefined groups [33]. As shown in Figure 1, the data points located in close proximity to the hyperplane are called support vectors, and lying at the centre of the margin is the optimal separating hyperplane that maximizes the separation gap for the training data points. A crucial aspect in SVM is the incorporation of kernel functions (radial basis function, polynomial, Fisher, and Bayesian) which play an important role in transforming non-linear decision surface of a lower dimensional space to a linear equation of a higher dimensional space. The equation of the hyperplane in ‘z’ dimensions for a given training dataset ‘*D*’ is expressed as follows:

(3) $$D=\{(x_{j},y_{j}),j=1,2,\ldots ,z|y_{j}\in \{+1,-1,x_{j}\in R^{n}\}$$

can be determined by:

(4) $$f\left(x\right)=\sum_{j=1}^{Z}{z_{j}\cdot y}_{j}\cdot x_{j}$$

where $x_{j}$ represents the input expressed as an n-dimensional real vector, $y_{j}$ is the output whose value is either 1 or −1, and $z_{j}$ is an SVM-generated multiplier. Once the hyperplane is established, the new input q is categorized using Equation (5):

(5) $$d\left(q\right)=sgn\left(\sum_{j=1}^{z}z_{j}\cdot y_{j}\cdot K\left(x_{j},x\right)+B\right)$$

where $K\left(x_{j},x\right)$ represents the kernel function, and B is the bias. The function maps the input dataset to a set of features as shown in Figure 1.

### 3.2. Feed-Forward Neural Networks (FFNNs)

**Figure 2:**
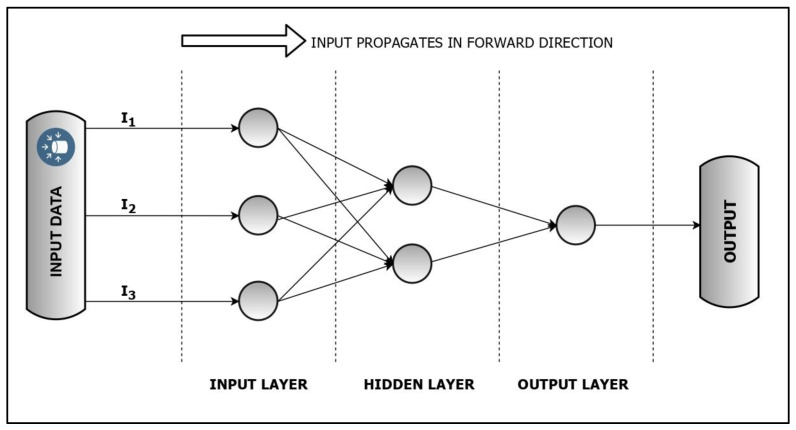
Schematic representation of an FFNN.

Feed-forward neural networks are one of the most basic forms of neural networks where the information propagation is only in the forward direction (no back-loops) [34]. The network architecture is composed of many simple processing elements known as neurons, which are generally arranged into a sequence of three fully connected layers, i.e., an input layer, a hidden layer, and an output layer. A schematic diagram of an FFNN is shown in Figure 2. Each neuron in a layer is conjoined in a unidirectional network with the help of weighted pathways called interconnections. The strength of these interconnections between the neurons corresponds to the adaptable synaptic weights. These weights multiplied by the incoming signals (inputs) are first summed and then transferred through an activation function to generate the final outcome (Equations (6) and (7)).

(6) $$T_{net}=\sum_{j=1}^{n}Y_{j}\cdot w_{j}+ꞵ$$

(7) $$T=f(T_{net})$$

where $T_{net}$ is the summation of the weighted inputs, $f(T_{net})$ is the non-linear activation function, $Y_{j}$ is the input neuron, $w_{j}$ is the weight coefficient, and ꞵ is the bias.

Further, the simulated observations of the network are compared with the actual observations, and the network error is computed using Equation (3)

(8) $$E_{r}=\frac{1}{2}\cdot \sum_{j=1}^{k}{\left(Y_{j}-O_{j}\right)}^{2}$$

where $E_{r}$ represents the error between the predicted and the observed value, and $O_{j}$ is the observed value of *j*th neuron.

### 3.3. Multiple Linear Regression

Multiple linear regression (MLR) is a generalized linear technique which attempts to model the association between the target variable and the predictor variable using linear combinations of the latter. The technique leads to a clearer and more precise understanding of the relationship between each individual predictor with the target and the relationships between the input predictors themselves. MLR is one of the most intuitive forecasting approaches, which presents several advantages such as lower computational cost, simple model structures, and parsimonious input data requirements in comparison with the physical models [35]. A general MLR model can be developed using the following equation:

(9) $$T=Y_{0}+Y_{1}X_{1}+Y_{2}X_{2}+\ldots +Y_{n}X_{n}+Er$$

where *T* denotes the dependent variable or the target, *Y_i_* (I = 0, 1, 2…n) represents the regression coefficients, *X_i_* is the independent variable, Y_0_ is a constant value (intercept), and *E_r_* is the error term.

### 3.4. Spotted Hyena Optimizer

The spotted hyena optimizer (SHO) is a state-of-the-art meta-heuristic optimization technique based on the foraging behaviour of spotted hyenas in nature [36]. The technique presents better outcomes for complex nonlinear problems and proves to be an efficient constraint-handling method in comparison with other algorithms [37]. The SHO mathematically depicts the hunting behaviour of spotted hyenas, which consists of four key steps, i.e., encircling, hunting, attacking, and searching for prey via a set of empirical equations. The following sections present the set of equations used to describe the hunting behaviour of spotted hyenas.

#### 3.4.1. Encircling

The spotted hyenas encircle the prey (current best solution) and, based on the prey’s location, they update their positions to acquire the target. The encircling behaviour in SHO is represented by the following set of equations:

(10) $$L_{h}=\left|C_{1}\cdot Y_{p}\left(t\right)-Y\left(t\right)\right|$$

(11) $$Y\left(t+1\right)=Y_{p}\left(t\right)-C_{2}\cdot L_{h}$$

where $L_{h}$ represents the distance a spotted hyena needs to cover to reach its prey, t is the ongoing iteration, $Y_{p}\left(t\right)$ represents the location of the prey, $Y\left(t\right)$ is the spotted hyena location, and $C_{1}$ and $C_{2}$ are the coefficients computed using Equations (12)–(14):

(12) $$C_{1}=2\cdot v_{1}$$

(13) $$C_{2}=2h\cdot v_{2}-h$$

(14) $$h=5-(Iteration*(5/{Max}_{iteration}))$$

where Iteration = 0, 1, 2,…, ${Max}_{iteration}$.

Here, *h* is linearly decreased from 5 to 0 over the course of iterations and $v_{1}$*,*$v_{2}$ are vectors of random numbers that are generated in the range [0, 1].

#### 3.4.2. Hunting

The hunting behaviour of the SHO is modelled using the following equations:

(15) $$L_{h}=\left|C_{1}\cdot Y_{h}\left(t\right)-Y_{k}\right|$$

(16) $$Y_{k}=C_{1}-C_{2}\cdot L_{h}$$

(17) $$N_{h}=Y_{k}+Y_{k+1}\ldots Y_{k+N}$$

where $Y_{h}$ is the first best-spotted hyena’s position, $Y_{k}$ represents the other hyena’s position, and *N* denotes the number of spotted hyenas, which may be further computed as:

(18) $$N={count}_{nos}\left(Y_{h},Y_{h+1},Y_{h+2}\ldots {(Y}_{h+M})\right)$$

where *M* is a randomly generated number whose value lies within [0.5, 1], *nos* is the number of solutions, and $N_{h}$ denotes the batch of *N* optimal responses.

#### 3.4.3. Attacking

Considering the above-mentioned equations, the attacking behaviour of the SHO can be represented as follows:

(19) $$Y\left(t+1\right)=\frac{N_{h}}{N}$$

where $Y\left(t+1\right)$ saves the best solution (hyena position) and helps in the position updation of others. 

#### 3.4.4. Searching

The search for prey depends on the location of the spotted hyena group represented by the vector $N_{h}$. To hunt effectively, these spotted hyenas must spread out and move away from each other. In the case of the SHO algorithm, the vector $C_{2}$ is varied randomly with values >1 or <−1, which allows the search factors (hyenas) to move further from the prey. Moreover, these factors tend to move towards the prey when the value of $\left|C_{2}\right|$ > 1. This process helps the SHO to show more randomization and acquire global optima.

### 3.5. Model Development

In this study, several AI models were established, such as RF, SVR, ANN, and a hybrid model (SVR–SHO) including the incorporation of SVR and the hyena algorithm. Furthermore, a statistical model such as MLR is also used in this study. In this study, a total of 50 experimental samples were utilized for model construction. Out of these, 33 samples (66%) were randomly selected for training the models, while the remaining 17 samples (34%) were reserved for validation purposes. Detailed statistical descriptions for both the training and testing data can be found in Table 1.

**Figure 3:**
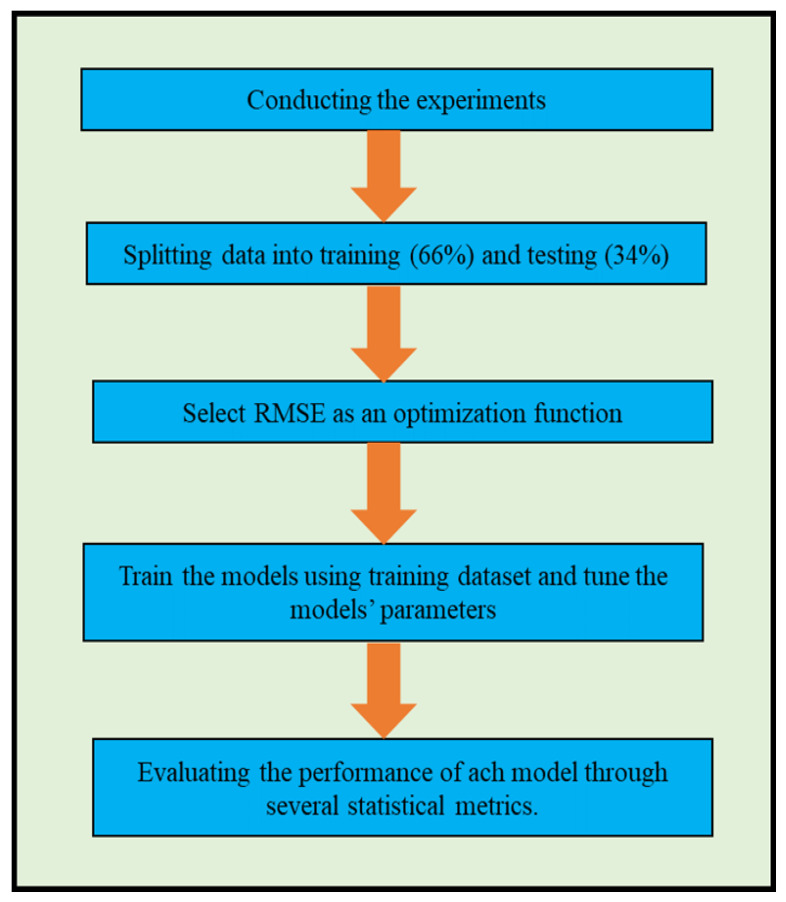
A flowchart showing the primary steps for models’ development.

The RMSE criteria used the optimization function. The hyperparameters of single models such as ANN, RF, and SVR have been selected via the trial-and-error method. However, the hyena algorithm was employed in this study to tune the hyperparameters of the SVR (i.e., kernel parameters, box constraints, and epsilon coefficients) [33,38] that have a significant impact on the model accuracy and performance. The model parameters that reduce the value of *RMSE* throughout the training phase will be used to create and evaluate the comparable model. MATLAB was used to establish all the models in this study. Figure 3 shows the main processes of developing the models.

In order to assess the suggested models, several statistical fitting indicators were used, such as correlation coefficient (*R*), coefficient of determination (*R*^2^), root mean square error (*RMSE*), mean absolute error (*MAE*), mean absolute percentage error (*MAPE*%), and the Willmott index of agreement (*WI*). These indicators are usually used to evaluate the performance of AI-based models [39] and can provide a deep insight into the accuracy of the predicted results [40]. The mathematical expressions of these indicators are provided as below [41,42,43,44,45]:

(20) $$R=\frac{\sum_{i=1}^{n}\left[\left(X_{{obs}_{i}}-\bar{X_{obs}}\right)\left(X_{{pred}_{i}}-\bar{X_{pred}}\right)\right]}{\sqrt{\sum_{i=1}^{n}{\left(X_{{obs}_{i}}-\bar{X_{obs}}\right)}^{2}\sum_{i=1}^{n}{\left(X_{{pred}_{i}}-\bar{X_{pred}}\right)}^{2}}}$$

(21) $$R^{2}=1-\frac{\sum_{i=1}^{n}(X_{{obs}_{i}}-X_{{pred}_{i}}{)}^{2}\;}{\sum_{i=1}^{n}{\left(X_{{pred}_{i}}-\bar{X_{pred}}\right)}^{2}}$$

(22) $$RMSE=\sqrt{\frac{1}{n}\sum_{i=1}^{n}{\left(X_{{obs}_{i}}-X_{{pred}_{i}}\right)}^{2}}$$

(23) $$MAE=\frac{1}{n}\sum_{i=1}^{n}\left|X_{{obs}_{i}}-X_{{pred}_{i}}\right|$$

(24) $$WI=1-\frac{\sum_{i=1}^{n}(X_{{obs}_{i}}-X_{{pred}_{i}}{)}^{2}}{\sum_{i=1}^{n}(\left|X_{{pred}_{i}}-\bar{X_{obs}}\right|{+\left|X_{{obs}_{i}}-\bar{X_{obs}}\right|)}^{2}}$$

(25) $$MAPE\%=\frac{100}{n}\sum_{i=1}^{n}\frac{\left|X_{{obs}_{i}}-X_{{pred}_{i}}\right|\;}{X_{{obs}_{i}}}$$

where $X_{{obs}_{i}}$ and $X_{{pred}_{i}}$ are the actual and predicted pressure flux of the ith sample, while $\bar{X_{obs}}$ and $\bar{X_{pred}}$ are the average values of actual and predicted pressure flux, and *n* is the total sample numbers. 

## 4. Results and Discussion

### 4.1. Experimental Results

**Figure 4:**
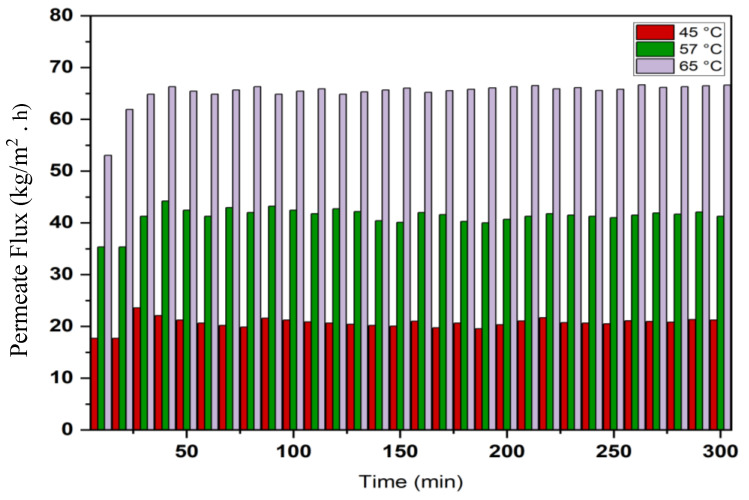
Flux variation with time at different feed temperatures, 0.6 L/min feed flow rate, 12.7 kPa (abs) absolute pressure, 35 g/L feed solution.

**Figure 5:**
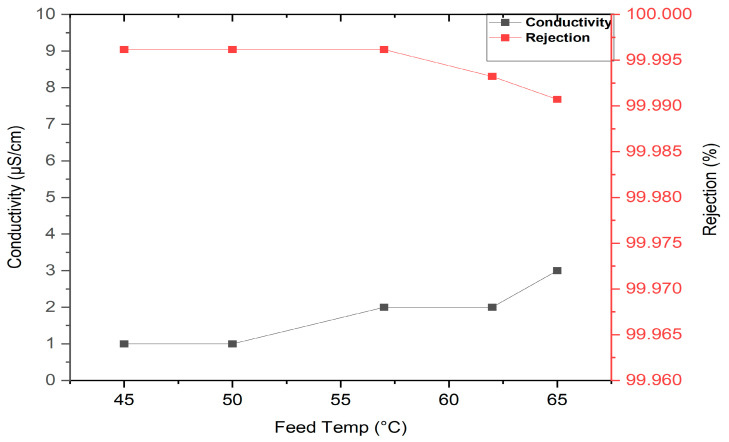
Effect of feed temperature on permeate conductivity and salt rejection for solution at 35 g/L, 0.6 L/min feed permeate flux, and 12.7 kPa (abs) absolute pressure.

**Figure 6:**
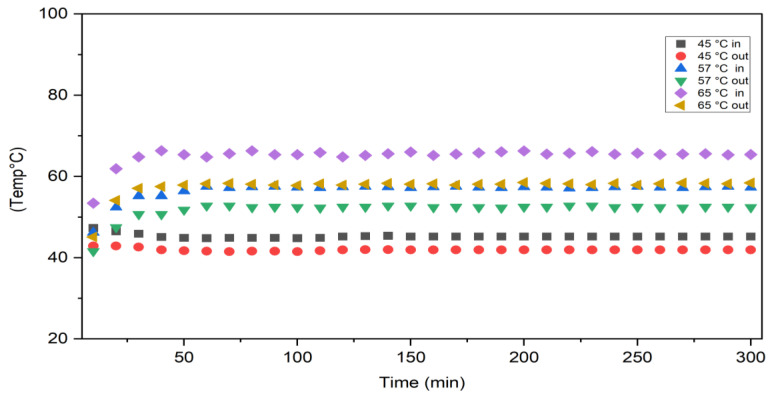
Inlet and retentate temperature behaviour of the VMD system over 300 min operation.

Permeate flux increases with increasing feed temperature as shown in Figure 4 because the actual driving force for MD is the vapour pressure difference across the membrane, which increases with increasing temperature. It can be noticed that the permeation flux was required for about 20–30 min to reach a steady state, then the flux was approximately constant until 300 min into the VMD process, indicating that there were no membrane-fouling effects since the module was washed after each run with distilled water. The effects of feed temperature on permeate conductivity and salt rejection for a solution of 35 g/L NaCl is illustrated in Figure 5. Permeate conductivity increased slightly with increasing feed temperature. This observation shows that temperature has a minor effect on the membrane pore-wetting process, and permeate conductivity ranged below 10 µS/cm with salt rejection achieved at about 99.99%. The conductivity of permeate for a concentration of 30 g/L NaCl was between 1.1–8.5 µS/cm at temperatures ranging between 60 and 80 °C. Figure 6 shows the behaviour of inlet and outlet feed temperatures with time for the VMD system during operation at 300 min at different feed temperatures. This figure also shows that the system required approximately 20–30 min to reach a steady state for the inlet and outlet feed temperatures. 

**Figure 7:**
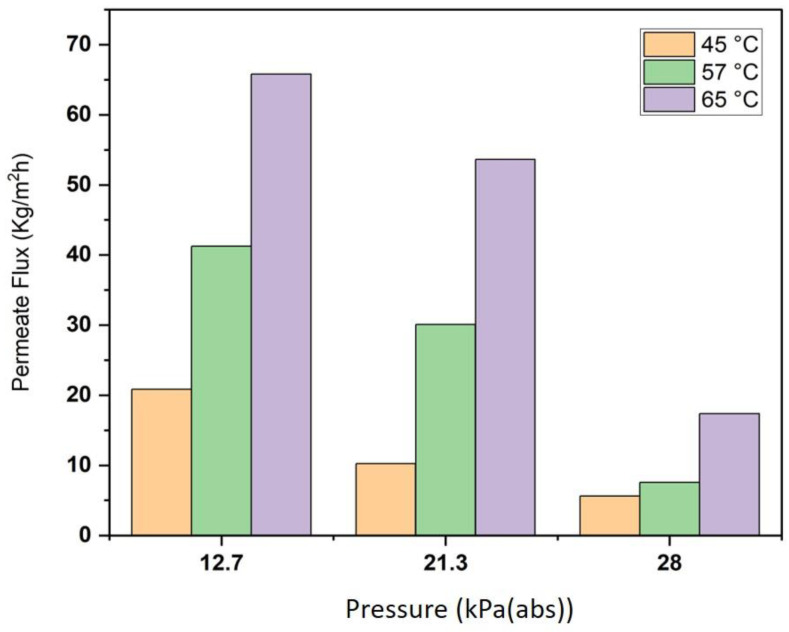
Effect of absolute pressure on permeate flux for salt solution at 35 g/L, 0.6 L/min feed permeate flux, and different feed temperatures.

The effects of absolute pressure (in a vacuum zone) on permeate flux at different feed temperatures are shown in Figure 7. The absolute pressure varied from 12.7 to 28 kPa (abs) at different feed temperatures (45, 57 and 65 °C) and permeate salt concentrations were maintained constant at 0.6 L/min and 35 g/L, respectively. It was found that permeate flux considerably decreases with the increase at the permeate side. The permeate flux declined by about 15–48 kg/m^2^ h when the absolute pressure increased from 12.7 to 28 kPa (abs) at different feed temperatures, which indicates the obvious effect of absolute pressure on MD flux. The influence of air in the membrane pores on the water vapour diffusion through the pores can be neglected in VMD; thus, the conduction heat transfer across the membrane can be neglected, and this leads to an increase in permeate flux. 

### 4.2. Artificial Intelligent-Based Models

In this section, the performance of the proposed models was evaluated via both the training and testing phases using various statistical matrices and graphical representations. The performance of MLR, ANN, SVR, and SVR−SHO during the training phase is summarized in Table 1. It can be observed from Table 2 that the SVR optimized with SHO meta-heuristic algorithm (SVR−SHO) provides the best performance in flux pressure (FP) prediction by producing fewer prediction errors ($MAE\approx 2.262\frac{Kg}{m^{2}.h}$, $RMSE\approx 6.330\frac{Kg}{m^{2}.h}$, and MAPE≈0.149) and providing the highest prediction accuracy (R≈0.946, and WI≈0.971). Moreover, the results showed that the ANN model performs significantly poorly, producing a high rate of prediction errors ($MAE\approx 8.238\frac{Kg}{m^{2}.h}$, RMSE≈15.974 $\frac{Kg}{m^{2}.h}$, and MAPE≈0.684) and lower prediction accuracy (R≈0.704, and WI≈0.834). The results showed the superiority of the SHO meta-heuristic algorithm in decreasing prediction error (76.562% for MAE, 47.386% for RMSE, and 71.673 % for MAPE) and increasing the prediction accuracy (9.364% for R, 19.141 for WI) for the vanilla SVR model. On the other hand, the MLR model outperformed the vanilla SVR model in FP prediction by producing fewer prediction errors ($MAE\approx 60554\frac{Kg}{m^{2}.h}$, $RMSE\approx 18.607\frac{Kg}{m^{2}.h}$, and MAPE≈0.319) and higher prediction accuracy (R≈0.894, and WI≈0.942). Overall, the SVR−SHO model reduced the prediction errors in terms of MAE≈65.487−76.561%, RMSE≈26.454−60.373%, and MAPE≈53.321−78.232%. Meanwhile, it improved prediction accuracy by ≈5.866−34.496% in terms of R and ≈3.111−19.155% in terms of WI compared to the other models, which indicates the high capability of the model to capture the behaviour of FP.

The results of the training phase reflect the learning ability of the proposed models. To this end, it is essential to test the performance of the proposed models during the testing phase. The primary motivation behind assessing the proposed models during the testing phase is that the proposed models receive only the input parameters in the testing phase, whereas in the training phase, models receive both input parameters and corresponding values. It also showed the potency of the proposed models as a predictive tool [1]. In this regard, Table 3 shows the performance of the proposed models during the testing phase. According to Table 3, the SVR−SHO model still provides robust prediction by producing fewer prediction errors ($MAE\approx 3.278\frac{Kg}{m^{2}.h},RMSE\approx 3.931\frac{Kg}{m^{2}.h},$ and MAPE≈11.5%) and producing the highest prediction accuracy (R≈0.971, and WI≈0.983), which is consistent with the findings from the training phase. On the other hand, the vanilla SVR model showed a significantly poor performance, producing a high error rate $(MAE\approx 10.087\frac{Kg}{m^{2}.h}$, $RMSE\approx 11.292\frac{Kg}{m^{2}.h}$ and MAPE≈34.9%) and lower prediction accuracy (R≈0.801 and WI≈0.709), which indicates that even though the error measurement in the training phase is acceptable, it does not imply that the model will perform better in the testing phase. Table 2 also shows the superiority of the SHO algorithm in the SVR–SHO hybrid model by reducing the error of the vanilla SVR model by ≈67.504, 65.189, and 66.925% in terms of MAE, RMSE, and MAPE, respectively. Furthermore, the hybrid model enhanced the vanilla model’s prediction accuracy by 21.153 and 38.672% in terms of *R* and *WI*, respectively.

The results also showed that despite the poor performance of the ANN model during the training phase, nevertheless, regarding the testing phase, the ANN model showed a significant improvement since the prediction error was reduced by 16.906, 43.386, and 63.475% for MAE, RMSE, and MAPE, respectively, and the prediction accuracy increased by 28.187 and 10.975% for *R* and *WI*, respectively. Moreover, the ANN model outperformed both SVR and MLR models during the testing phase.

**Figure 8:**
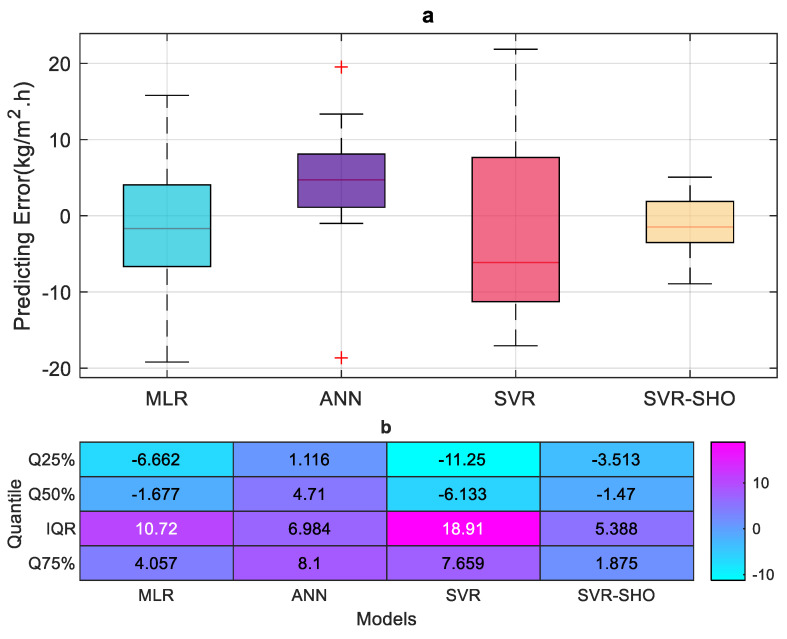
Boxplot of the prediction errors by the proposed models during the testing phase. (**a**) Boxplot diagram, (**b**) Quantile range of predicting error for each model (Q75, Q50, Q75, IQR).

Several graphical presentations have been created for better performance assessment of the proposed models during the testing phase. Figure 8a presents a boxplot showing the prediction error distribution for the proposed models. The boxplot includes a box drawn between the first quartile (Q25%) and the third quartile (Q75%), which is also known as the interquartile range $\left(IQR\right)$. The horizontal line in the box represents the median of the data, also known as the second quartile (Q50%). The extended lines from the first and third quartiles are called the whiskers, which indicate the variability outside these quartiles, while the outliers are drawn as individual points. According to Figure 8a,b, it can be seen that the SVR−SHO model provides fewer IQR values (IQR=5.388) which indicates that the SVR–SHO model generates minor prediction errors. On the other hand, the distribution of prediction errors in the vanilla SVR model is significantly high (IQR=18.91), which indicates the poor performance of the SVR in predicting FP. 

**Figure 9:**
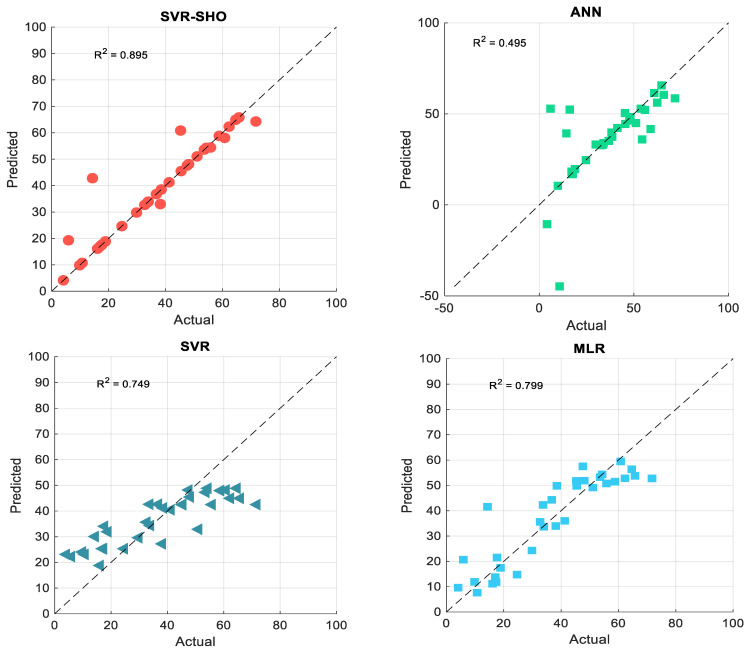
Scatter plot of actual and predicted FP values obtained via the proposed models and experimental data: training phase.

**Figure 10:**
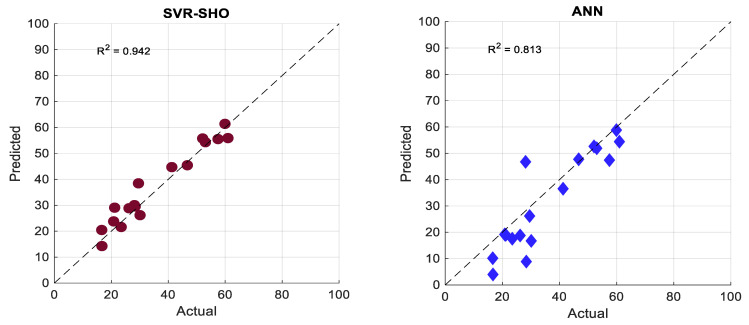

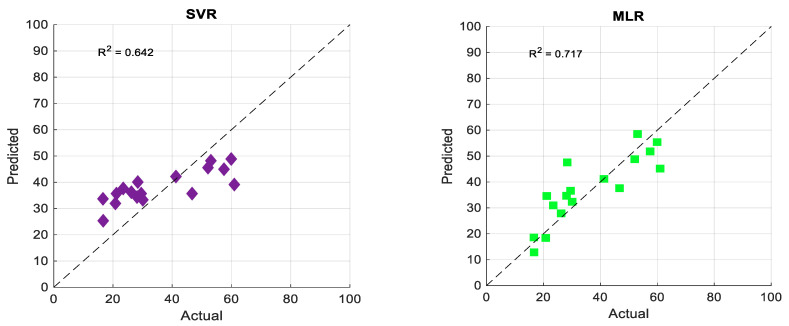
Scatter plot of actual and predicted FP values obtained via the proposed models and experimental data: testing phase.

Figure 9 shows the scatter plot that examines the proposed models’ prediction efficiency via the testing phase. It can be seen from Figure 9 and Figure 10 that the SVR−SHO model exhibits an excellent correlation between the actual and predicted FP values providing an $R^{2}$ value of 0.942. Meanwhile, the vanilla SVR model produces a significantly poor correlation between the actual and predicted FP values, with $R^{2}=0.642$. According to Figure 9 and Figure 10, the hybrid models demonstrated the highest R^2^ values during both the training and testing phases, surpassing the performance of other models.

To obtain further insight into the capacity of the proposed models to predict the FP value, the relative error diagram was constructed as shown in Figure 10 is used based on the testing phase. 

**Figure 11:**
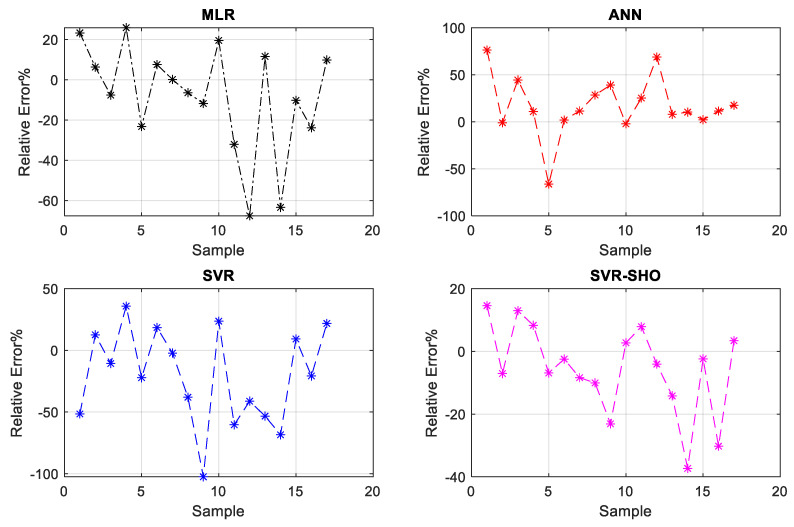
The relative error distribution diagrams of the proposed models during the testing phase.

According to Figure 11, the SVR−SHO model provides 80% of the dataset, with RE% ranging between (−20 to 20%). Meanwhile, three observation indicates an RE% of more than 20%. A possible explanation for this result might be that no related information was incorporated into the training data to replicate those three observations in their actual magnitude. Overall, the SVR−SHO model provides good results in terms of RE%. Conversely, the SVR model provides the poorest performance in terms of RE%.

**Figure 12:**
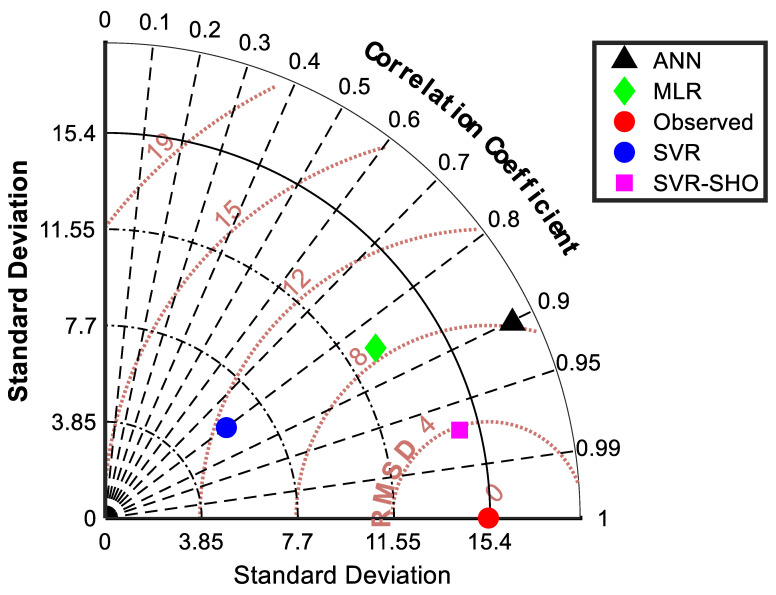
Graphical visualization of Taylor diagram for the proposed models.

The similarities between predicted and actual FP values were graphically presented in a Taylor diagram (Figure 12) for further assessment. The Taylor diagram can highlight the efficiency of the proposed models, where a series of points (models) can be visualized on a polar plot based on the correlation coefficient and the standard deviation. Furthermore, it calculates the ratio of variance in order to obtain the relative depth of the actual and predicted variations. Evidently, Figure 12 shows that the SVR−SHO model is closer to the observed value than the other models. Notably, the hyperparameters of all applied models are provided in Table 4.

The results clearly demonstrated that the hybrid model exhibited outstanding performance and yielded more precise results compared to traditional models such as ANN, MLR, and classical SVR. The enhancement in predictive capability can be attributed to the synergistic combination of two algorithms, namely SVR and SHO. SHO proved to be particularly effective in optimizing the hyperparameters of SVR, enabling the model to make robust predictions of permeate flux even in scenarios where the available data samples were limited. This effectiveness is likely due to SHO’s ability to efficiently solve optimization problems by striking a balance between exploration and exploitation, thus discovering global solutions [46]. Additionally, SHO is widely recognized for its simplicity, adaptability, and robustness, consistently delivering competitive results when compared to other metaheuristics. By thoroughly exploring and exploiting the search space, SHO is able to identify optimal solutions and find global optima even in complex problem environments, resulting in high-quality solutions that approach global optimality.

### 4.3. Impact of Operating Parameters on Flux

**Figure 13:**
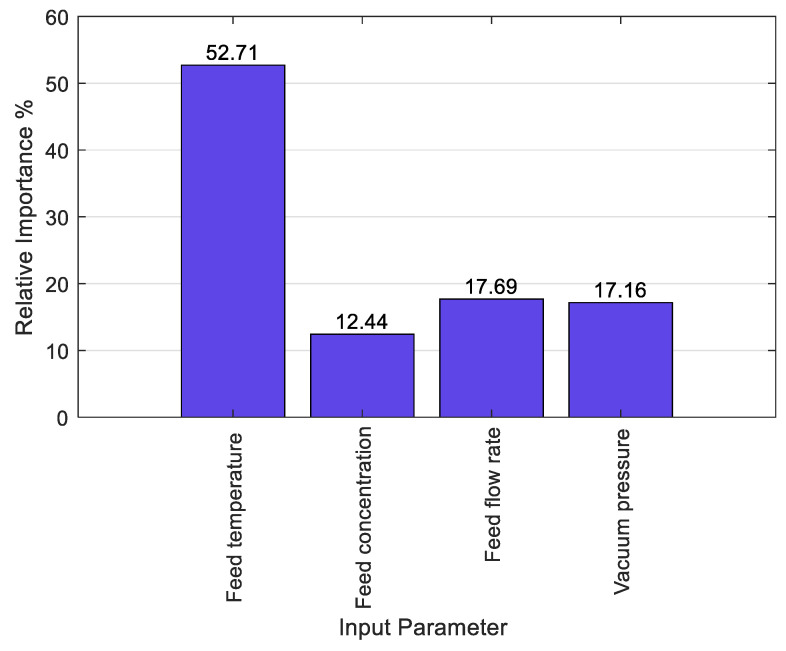
The relative importance of four input components using the SVR−SHO model.

Considering the high accuracy of the predictions obtained via the SVR−SHO model, we only focused on this model to interpret the FP data. For the SVR−SHO model, global sensitivity analysis (GSA) is adopted to obtain the relative importance of each input parameter. GSA is a method that is based on the Monte Carlo simulation approach used to prioritize the importance of parameters for a given modelling exercise. The technique is a variance-based approach which proportionally quantifies the uncertainty of each input parameter to the uncertainty of the model output. The fundamental principle behind the GSA is to fix all the input parameters to a known value, apart from the one parameter that needs to be computed, and then use the available formula to determine the output weight that corresponds to each of the input parameters [47]. In a practical sense, GSA can be applied to any supervised machine learning algorithm for regression tasks. Figure 13 shows the relative importance of four input components obtained via SVR−SHO combined with GSA. The obtained results showed that the feed temperature is the most impactful parameter regarding flux pressure, with a relative importance of 52.71%, followed by feed permeate flux and vacuum pressure with a relative importance of 17.69% and 17.16%, respectively. On the other hand, feed concentration has a minor influential effect on the results, with a relative importance of 12.44%.

## 5. Conclusions

A new hybrid model called SVR–SHO has been created to predict the flux pressure of VMD. This model combines the SHO algorithm and SVR and has been developed and tested using experimental data. The performance of SVR–SHO was compared to other commonly used machine learning models such as ANN, classical SVR, and a statistical model called MLR. The prediction results showed that the SVR–SHO model outperformed the other models, achieving high accuracy with an R of 0.94. Additionally, a global sensitivity analysis was conducted to interpret the predictive results, which revealed that the feed temperature was the most influential parameter affecting the flux pressure.

The results achieved with the SVR–SHO model and global sensitivity analysis underscore their potential as a valuable tool for more effectively studying membrane performance. By leveraging software-driven data and advanced modelling techniques such as SVR–SHO and global sensitivity analysis, valuable insights can be gained into the complex behaviour of membrane systems. Also, such approaches enable more informed decisions and optimized experimental setup, leading to improved efficiency and performance. Thus, it would be beneficial to further test the SVR–SHO model in more complex systems that involve hybrid membrane technologies and changing membrane properties with fouling accumulation emanating from different contaminants.

## Figures and Tables

**Table 1:** The statistical description of the used data.

Data	Parameter	Type	Mean	Minimum	Maximum	Standard Deviation
Training data	Feed temperature (°C))	Input	56.85	45.00	65.00	8.22
	Feed concentration (g/L)		51.52	0.00	100.00	32.12
	Feed permeate flux (L/min)		0.52	0.30	0.60	0.10
	Vacuum pressure (kPa (abs))		15.39	12.70	28.00	5.10
	Permeate flux (kg/m^2^ h)	Output	35.14	5.92	62.32	17.04
Testing data	Feed temperature (°C))	Input	59.83	50.00	65.00	6.21
	Feed concentration (g/L)		63.33	35.00	100.00	24.83
	Feed permeate flux (L/min)		0.55	0.40	0.60	0.08
	Vacuum pressure (kPa (abs))		12.70	12.70	12.70	0.00
	Permeate flux (kg/m^2^ h)	Output	47.49	23.47	71.72	19.32

**Table 2:** The performance of the proposed models during the training phase.

Models/Performance Indicator	MAE $\left(\frac{Kg}{m^{2}.h}\right)$	RMSE $\left(\frac{Kg}{m^{2}.h}\right)$	MAPE%	R	WI
MLR	6.554	8.607	31.9	0.894	0.942
ANN	8.238	15.974	68.4	0.704	0.834
SVR	9.651	12.031	52.6	0.865	0.815
SVR–SHO	2.262	6.330	14.9	0.946	0.971

**Table 3:** The performance of the proposed models during the testing phase.

Models/Performance Indicator	MAE $\left(\frac{Kg}{m^{2}.h}\right)$	RMSE $\left(\frac{Kg}{m^{2}.h}\right)$	MAPE%	R	WI
MLR	6.466	8.250	20.6	0.847	0.910
ANN	6.846	9.043	25.0	0.902	0.926
SVR	10.087	11.292	34.9	0.801	0.709
SVR–SHO	3.278	3.931	11.5	0.971	0.983

**Table 4:** Model hyperparameters.

Model	Hyperparameters
SVR–SHO	1. Kernel Scale is 0.8940
	2. Box Constraint is 2.2599
	3. Epsilon is 1 × 10^−4^
SVR	1. Kernel Scale is 1
	2. Box Constraint is 0.2684
	3. Epsilon is 0.0268
ANN	1. Hidden Layer is 8 2. Transfer Function is hyperbolic tangent sigmoid 3. Algorithm is Levenberg–Marquardt
SHO	1. Number of Search Agents (population) is 14 2. Maximum Number of Iterations is 100

## Data Availability

All data are available upon request.

## References

[1] Aljumaily M.M., Alayan H.M., Mohammed A.A., Alsaadi M.A., Alsalhy Q.F., Figoli A., Criscuoli A. (2022). The influence of coating super-hydrophobic carbon nanomaterials on the performance of membrane distillation. Appl. Water Sci..

[2] Rohani R., Yusoff I.I., Khairul Zaman N., Mahmood Ali A., Rusli N.A.B., Tajau R., Basiron S.A. (2021). Ammonia removal from raw water by using adsorptive membrane filtration process. Sep. Purif. Technol..

[3] Aljanabi A.A.A., Mousa N.E., Aljumaily M.M., Majdi H.S., Yahya A.A., Al-Baiati M.N., Hashim N., Rashid K.T., Al-Saadi S., Alsalhy Q.F. (2022). Modification of Polyethersulfone Ultrafiltration Membrane Using Poly (terephthalic acid-co-glycerol-g-maleic anhydride) as Novel Pore Former. Polymers.

[4] Altaee A., Alhathal Alanezi A., Alqahs Alanezi Y., Alazmi R., Alsalhy Q., Sharif A. (2020). Enhancing Performance of the Membrane Distillation Process using Air Injection Zigzag System for Water Desalination. Desalination Water Treat..

[5] Abujazar M.S.S., Fatihah S., Kabeel A.E. (2017). Seawater desalination using inclined stepped solar still with copper trays in a wet tropical climate. Desalination.

[6] Mahdavi M., Mahvi A.H., Nasseri S., Yunesian M. (2011). Application of freezing to the desalination of saline water. Arab. J. Sci. Eng..

[7] Aljumaily M.M., Ali N.S., Mahdi A.E., Alayan H.M., AlOmar M., Hameed M.M., Ismael B., Alsalhy Q.F., Alsaadi M.A., Majdi H.S. (2022). Modification of Poly (vinylidene fluoride-co-hexafluoropropylene) Membranes with DES-Functionalized Carbon Nanospheres for Removal of Methyl Orange by Membrane Distillation. Water.

[8] Jamed M.J., Alhathal Alanezi A., Alsalhy Q.F. (2019). Effects of embedding functionalized multi-walled carbon nanotubes and alumina on the direct contact poly (vinylidene fluoride-co-hexafluoropropylene) membrane distillation performance. Chem. Eng. Commun..

[9] Madalosso H.B., Machado R., Hotza D., Marangoni C. (2021). Membrane Surface Modification by Electrospinning, Coating, and Plasma for Membrane Distillation Applications: A State-of-the-Art Review. Adv. Eng. Mater..

[10] Alsalhy Q.F., Rashid K.T., Ibrahim S.S., Ghanim A.H., Van der Bruggen B., Luis P., Zablouk M. (2013). Poly (vinylidene fluoride-co-hexafluoropropylene)(PVDF-co-HFP) hollow fiber membranes prepared from PVDF-co-HFP/PEG-600Mw/DMAC solution for membrane distillation. J. Appl. Polym. Sci..

[11] Francis L., Ahmed F.E., Hilal N. (2022). Electrospun membranes for membrane distillation: The state of play and recent advances. Desalination.

[12] Alsalhy Q.F., Ibrahim S.S., Hashim F.A. (2018). Experimental and theoretical investigation of air gap membrane distillation process for water desalination. Chem. Eng. Res. Des..

[13] Safi N.N., Ibrahim S.S., Zouli N., Majdi H.S., Alsalhy Q.F., Drioli E., Figoli A. (2020). A systematic framework for optimizing a sweeping gas membrane distillation (SGMD). Membranes.

[14] Ghaffour N., Soukane S., Lee J.-G., Kim Y., Alpatova A. (2019). Membrane distillation hybrids for water production and energy efficiency enhancement: A critical review. Appl. Energy.

[15] Mohanadas D., Nordin P.M.I., Rohani R., Dzulkharnien N.S.F., Mohammad A.W., Mohamed Abdul P., Abu Bakar S. (2022). A Comparison between Various Polymeric Membranes for Oily Wastewater Treatment via Membrane Distillation Process. Membranes.

[16] Andrés-Mañas J., Ruiz-Aguirre A., Acién F., Zaragoza G. (2020). Performance increase of membrane distillation pilot scale modules operating in vacuum-enhanced air-gap configuration. Desalination.

[17] Alayan H.M., Aljumaily M.M., Alsaadi M.A., Mjalli F.S., Hashim M.A. (2021). A review exploring the adsorptive removal of organic micropollutants on tailored hierarchical carbon nanotubes. Toxicol. Environ. Chem..

[18] Alayan H., Aljumaily M.M., Alsaadi M.A., Hashim M.A. (2020). Probing the Effect of Gaseous Hydrocarbon Precursors on the Adsorptive Efficiency of Synthesized Carbon-Based Nanomaterials. J. Eng. Res..

[19] Ahmad N.N.R., Ang W.L., Leo C.P., Mohammad A.W., Hilal N. (2021). Current advances in membrane technologies for saline wastewater treatment: A comprehensive review. Desalination.

[20] Arumugham T., Kaleekkal N.J., Gopal S., Nambikkattu J., Rambabu K., Aboulella A.M., Wickramasinghe S.R., Banat F. (2021). Recent developments in porous ceramic membranes for wastewater treatment and desalination: A review. J. Environ. Manag..

[21] Ahmed F.E., Hashaikeh R., Diabat A., Hilal N. (2019). Mathematical and optimization modelling in desalination: State-of-the-art and future direction. Desalination.

[22] Ibrahim S.S., Alsalhy Q.F. (2013). Modeling and simulation for direct contact membrane distillation in hollow fiber modules. AIChE J..

[23] Dong Y., Dai X., Zhao L., Gao L., Xie Z., Zhang J. (2021). Review of transport phenomena and popular modelling approaches in membrane distillation. Membranes.

[24] Papapicco D., Demo N., Girfoglio M., Stabile G., Rozza G. (2022). The Neural Network shifted-proper orthogonal decomposition: A machine learning approach for non-linear reduction of hyperbolic equations. Comput. Methods Appl. Mech. Eng..

[25] Maslahati Roudi A., Chelliapan S., Wan Mohtar W.H.M., Kamyab H. (2018). Prediction and optimization of the fenton process for the treatment of landfill leachate using an artificial neural network. Water.

[26] Orrù G., Monaro M., Conversano C., Gemignani A., Sartori G. (2020). Machine learning in psychometrics and psychological research. Front. Psychol..

[27] Khayet M., Cojocaru C. (2012). Artificial neural network modeling and optimization of desalination by air gap membrane distillation. Sep. Purif. Technol..

[28] Yusuf A., Sodiq A., Giwa A., Eke J., Pikuda O., De Luca G., Di Salvo J.L., Chakraborty S. (2020). A review of emerging trends in membrane science and technology for sustainable water treatment. J. Clean. Prod..

[29] Nasir T., Asmaela M., Zeeshana Q., Solyalib D. (2020). Applications of machine learning to friction stir welding process optimization. J. Kejuruter..

[30] Jawad J., Hawari A.H., Javaid Zaidi S. (2021). Artificial neural network modeling of wastewater treatment and desalination using membrane processes: A review. Chem. Eng. J..

[31] Hameed M., Sharqi S.S., Yaseen Z.M., Afan H.A., Hussain A., Elshafie A. (2017). Application of artificial intelligence (AI) techniques in water quality index prediction: A case study in tropical region, Malaysia. Neural Comput. Appl..

[32] Sheikh Khozani Z., Hosseinjanzadeh H., Wan Mohtar W.H.M. (2019). Shear force estimation in rough boundaries using SVR method. Appl. Water Sci..

[33] Alomar M.K., Khaleel F., Aljumaily M.M., Masood A., Razali S.F.M., AlSaadi M.A., Al-Ansari N., Hameed M.M. (2022). Data-driven models for atmospheric air temperature forecasting at a continental climate region. PLoS ONE.

[34] Titah H.S., Halmi M.I.E.B., Abdullah S.R.S., Hasan H.A., Idris M., Anuar N. (2018). Statistical optimization of the phytoremediation of arsenic by Ludwigia octovalvis- in a pilot reed bed using response surface methodology (RSM) versus an artificial neural network (ANN). Int. J. Phytoremediation.

[35] Fang T., Lahdelma R. (2016). Evaluation of a multiple linear regression model and SARIMA model in forecasting heat demand for district heating system. Appl. Energy.

[36] Dragoi E.N., Dafinescu V. (2021). Review of Metaheuristics Inspired from the Animal Kingdom. Mathematics.

[37] Dhiman G., Kumar V. (2019). Spotted hyena optimizer for solving complex and non-linear constrained engineering problems. Harmony Search and Nature Inspired Optimization Algorithms.

[38] Hameed M.M., Razali S.F.M., Mohtar W.H.M.W., Rahman N.A., Yaseen Z.M. (2023). Machine learning models development for accurate multi-months ahead drought forecasting: Case study of the Great Lakes, North America. PLoS ONE.

[39] Adnan R.M., Mostafa R.R., Dai H.-L., Heddam S., Masood A., Kisi O. (2023). Enhancing accuracy of extreme learning machine in predicting river flow using improved reptile search algorithm. Stoch. Environ. Res. Risk Assess..

[40] Hameed M.M., AlOmar M.K., Al-Saadi A.A.A., AlSaadi M.A. (2022). Inflow forecasting using regularized extreme learning machine: Haditha reservoir chosen as case study. Stoch. Environ. Res. Risk Assess..

[41] Hameed M.M., Khaleel F., AlOmar M.K., Mohd Razali S.F., AlSaadi M.A. (2022). Optimising the Selection of Input Variables to Increase the Predicting Accuracy of Shear Strength for Deep Beams. Complexity.

[42] AlOmar M.K., Hameed M.M., Al-Ansari N., AlSaadi M.A. (2020). Data-Driven Model for the Prediction of Total Dissolved Gas: Robust Artificial Intelligence Approach. Adv. Civ. Eng..

[43] Hameed M.M., AlOmar M.K., Mohd Razali S.F., Kareem Khalaf M.A., Baniya W.J., Sharafati A., AlSaadi M.A. (2021). Application of Artificial Intelligence Models for Evapotranspiration Prediction along the Southern Coast of Turkey. Complexity.

[44] Masood A., Ahmad K. (2022). Data-driven predictive modeling of PM2.5 concentrations using machine learning and deep learning techniques: A case study of Delhi, India. Environ. Monit. Assess..

[45] Masood A., Ahmad K. (2023). Prediction of PM2.5 concentrations using soft computing techniques for the megacity Delhi, India. Stoch. Environ. Res. Risk Assess..

[46] Ghafori S., Gharehchopogh F.S. (2022). Advances in Spotted Hyena Optimizer: A Comprehensive Survey. Arch. Comput. Methods Eng..

[47] Chen S., Gu C., Lin C., Wang Y., Hariri-Ardebili M.A. (2020). Prediction, monitoring, and interpretation of dam leakage flow via adaptative kernel extreme learning machine. Measurement.

